# Microbial diversity in earthen site of exhibition Hall of pit no. 1 at the terracotta warriors Museum in Emperor Qinshihuang’s mausoleum site museum and its correlation with environmental factors

**DOI:** 10.3389/fmicb.2024.1378180

**Published:** 2024-09-20

**Authors:** Cen Wang, Lilong Hou, Nan Jiang, Yu Wang, Xiaofen Mao, Ping Zhou, Yin Xia, Yuanyuan Wang, Chuyue Chen, Xinyu Yang, Qiang Luo, Jiao Pan

**Affiliations:** ^1^Key Laboratory of Archaeomaterials and Conservation, Ministry of Education, Institute for Cultural Heritage and History of Science and Technology, University of Science and Technology Beijing, Beijing, China; ^2^Ministry of Education Key Laboratory of Molecular Microbiology and Technology, Department of Microbiology, College of Life Sciences, Nankai University, Tianjin, China; ^3^Institute of Applied Ecology, Chinese Academy of Sciences, Shenyang, China; ^4^Emperor Qinshihuang’s Mausoleum Site Museum, Key Scientific Research Base of Ancient Polychrome Pottery Conservation of State Administration of Culture Heritage, Shaanxi Xi’an, China

**Keywords:** the terracotta warriors museum, microbial diversity, earthen site, environmental factor, function of microorganisms

## Abstract

**Introduction:**

Earthen sites are essential cultural relic resources, and site museums are a fundamental component of China’s cultural heritage protection. The mausoleum of the Qin Shi Huang Emperor is one of the largest, most peculiar, and richest imperial tombs in the world. The exhibition hall of the burial pit No. 1 of the Terra Cotta Warriors is the earliest exhibition hall built and opened to the public. However, after years of excavation and open exhibitions, the earthen site of the Emperor Qinshihuang’s Mausoleum Site Museum has deteriorated to varying degrees due to changes in the modern environment. There is an urgent need to control microbial diseases and protect the earthen site.

**Methods:**

We analyzed the physical and chemical properties and bioindicators of the collected soil samples. We also established a metagenomic library and conducted a correlation analysis between microbial community composition and environmental factors. Cultivable fungi obtained from air and soil samples were identified, and allicin volatile gas fungistasis test was conducted.

**Result:**

Research has found that four different areas of the exhibition hall have different types of microbial diseases owing to their different environments. The main pathogenic fungi in earthen site may lead to potential microbial diseases that affect important cultural relics such as the Terra Cotta Warriors. *Penicillium*, *Aspergillus* and *Talaromyces* showed relatively specific growth in relation to environmental factors and showed a better raw growth advantage.Allicin gas had a inhibitory effect on 12 types of fungi, therefore allicin gas had a potent inhibitory effect on the growth of the most culturable fungal hyphae.

**Discussion:**

This study provides basic data for the study of microbial diversity in the exhibition hall of Pit No. 1 at the Terracotta Warriors Museum in Emperor Qinshihuang’s Mausoleum Site Museum. It provides a reference for future protection work, which is of great significance.

## 1 Introduction

Earthen sites are historical sites built with soil as the main building material and are important evidence of ancient human activity (Xiaoying et al., 2016; Kong et al., 2019; Xia Yin et al., 2023). There are nearly 1, 000 earthen sites in China according to the statistics of China’s early key cultural relics protection institutions (Luo Xilian et al., 2018). Meanwhile, more than 80 Chinese earth sites are on the World Heritage List (2019) published by UNESCO (Briseghella et al., 2020). However, these earthen sites are often extremely fragile, and many are being degraded as a result of inappropriate excavation processes and unsuitableprotective environments (Richards et al., 2020; Yumin et al., 2020). Due to the diversity of topography, hydrological environments, climatic environments and built forms of earthen sites, the protection of different earthen sites should vary according to their characteristics (Chonggen et al., 2020). Site museums are a fundamental part of cultural heritage conservation in China (Gu et al., 2013). For example, a cultural museum has been built on the site in order to protect the Jinsha Site Museum (Zhu and Kan, 2009; Wei et al., 2019). The same is true of the Emperor Qinshihuang’s Mausoleum Site Museum. The protection of the burial pits of the Terracotta Warriors belongs to the *in-situ* protection of the site, which is the protection of the earthen site by most of the site museums in China (Zhang et al., 2009). Prior to excavation, the cultural relic artifacts as well as the earthen site were rarely affected by the external atmospheric environment and remained in a very stable preservation environment for many years (Zhuangbo et al., 2022). However, exposed sites are susceptible to damage from the surrounding environment, such as changes in temperature and humidity (Xilian et al., 2019; He et al., 2021; Zhang et al., 2022). The impact of human activities such as visits and archaeological research on earthen sites can lead to destabilization of microbial populations. Therefore the protection of soil sites should be emphasized.

Currently, research focuses on the effects of physical and chemical factors on the degradation of earthen sites (Ma et al., 2020; Xudong et al., 2020; Germinario and Oguchi, 2021). Instead, little attention has been paid to the interaction of biological factors, especially microorganisms, in the degradation of earthen sites. Characterizing the microbial communities living in artifacts, particularly the identification of the most destructive microorganisms, will provide a basis for standardized microbiological control of protected earthen sites (Yang et al., 2020). Currently, only 1% of microorganisms can be cultured under laboratory conditions, so that methods for genome characterization is an important tool for determining the diversity and composition of microbial communities and functions (Sauro et al., 2018), such as metagenomics data assembly and analyses. It is an experimentally validated method for studying microbial communities in environments provides a powerful method, and has been successfully used to determine the composition and diversity of microbial communities from different environments (Alam et al., 2021), such as samples from river mud, groundwater and soil (Elena et al., 2019; Zaouri et al., 2020; Jiaxian et al., 2021). He Jintao had use the metagenomics data assembly to analyze the assembly processes of microbial communities in stone biodeterioration of the West Lake UNESCO world heritage area in China (Jintao et al., 2022).

Understanding how environmental factors affect the soil microbial community can also be achieved at the same time as accurately understanding how the microbial community affects the earthen sites (Carini et al., 2020). For example, carbon and nitrogen in the soil may affect the growth and metabolism of microorganisms through a variety of direct or indirect effects, and even change the structure of the microbial community in the soil as a whole, while microorganisms may be involved in nitrogen metabolism and decomposition in the soil (Jiang et al., 2013). There are two sources of fungal diseases on the surface of soil sites. On the one hand, many of the soils were not specially treated during the construction process, and the earthen sites contain many fungal spores, on the other hand the dispersal of mold spores brought by fallout and visitor activities (Xingqun et al., 1999).These spores cause damage to the earthen sites when the museum soil is moist and at the right temperature, and the fungal spores utilize the organic matter in the soil as a nutrient resuscitation for survival. There are a number of earth sites have been affected by various microorganisms or have undergone microbial degradation. Microbial diseases have emerged at archaeological sites in Liangzhu City, and there is a strong correlation between microbial diversity and the physical and chemical properties of the soil. The presence of Acidibacter may cause the continuous degradation of organic matter in the soil, such that the soil loses its original structure, becomes loose, and easily falls off (Jing et al., 2019). With an increase in the degree of degradation of the Jinsha soil site, the soil microbial community and functional diversity also flourished, and many types of bacteria are involved in the soil degradation process (Tongtong et al., 2017). The microbial community on the surface of the Lidu wine cellar site was determined and analyzed, and its dominant fungi mainly include filamentous fungi and yeast (Wu Fasi et al., 2012). The corrosive fungi on the surface of the Western Zhou Tomb Site in Dahekou, Yicheng, Shanxi Province, mainly belong to Pseudomonas and Alternaria (Yin et al., 2013).

The Qin Shi Huang Emperor mausoleum, built from 247 to 208 BC, is one of the largest, most peculiar, and richest imperial mausoleums in the world. It fully reflects the artistic wisdom of the Han working people in ancient China more than 2000 years ago and is a precious resort for the Chinese nation. On March 4, 1961, the mausoleum of the Qin Shi Huang Emperor was announced by the State Council as the first batch of major historical and cultural sites to be protected at the national level. The Qin Shi Huang Mausoleum and the Terra Cotta Warriors Pit were approved by UNESCO for inclusion on the World Heritage List in December 1987. The No. 1 Pit exhibition hall is a typical semi-open museum. It is the first exhibition hall built and opened to the public at the Emperor Qinshihuang’s Mausoleum Site Museum and is the largest core exhibition hall.

However, after years of excavation and open exhibition, the earthen sites and unearthed cultural relics of the Emperor Qinshihuang’s Mausoleum Site Museum have also deteriorated to varying degrees due to changes in the modern environment, resulting in the ruins unable to maintain their original state (Qiang et al., 2021). A large number of microorganisms and various coarse and fine particles to gather in the exhibition hall of Pit 1. The aggregation of flocs promotes the adsorption of these microorganisms and particles on the surface of the site, causing the accumulation of dark sediment on the surface and leading to further destructive effects such as biological corrosion, biodegradation, and pigment pollution produced by microorganisms. Luo et al. isolated and identified four main fungi in flocs: Aspergillus niger, Aspergillus flavus, Penicillium oxalate, and *Alternaria alternata*. The diversity of fungal communities in the flocs was analyzed, with Alternaria and Aspergillus occupying an absolute advantage (Qiang et al., 2021). There is an urgent need to study and control microbial diseases and protect the earthen site of the Emperor Qinshihuang’s Mausoleum Site Museum.

Choosing plant-based antifungal agents from actual plants is safer and more environmentally friendly (Al-Qahtani et al., 2022). Plant volatile oil, which is widely found in roots, bark, stems, leaves, flowers, and fruits, is composed of terpenes, ketones, epoxides, esters, acids, alcohols, and sulfides and has good antibacterial, antifungal, antiviral, and antioxidant effects (Basile et al., 2009; Thotathil et al., 2023). Yang’s treatment with allicin interfered with the biosynthesis, glucose catabolism, and oxidative stress of the cell membrane and cell wall of Trichosporon alfredii, proving that allicin had a favorable effect on fungal inhibition (Yang et al., 2023).

**Figure 1:**
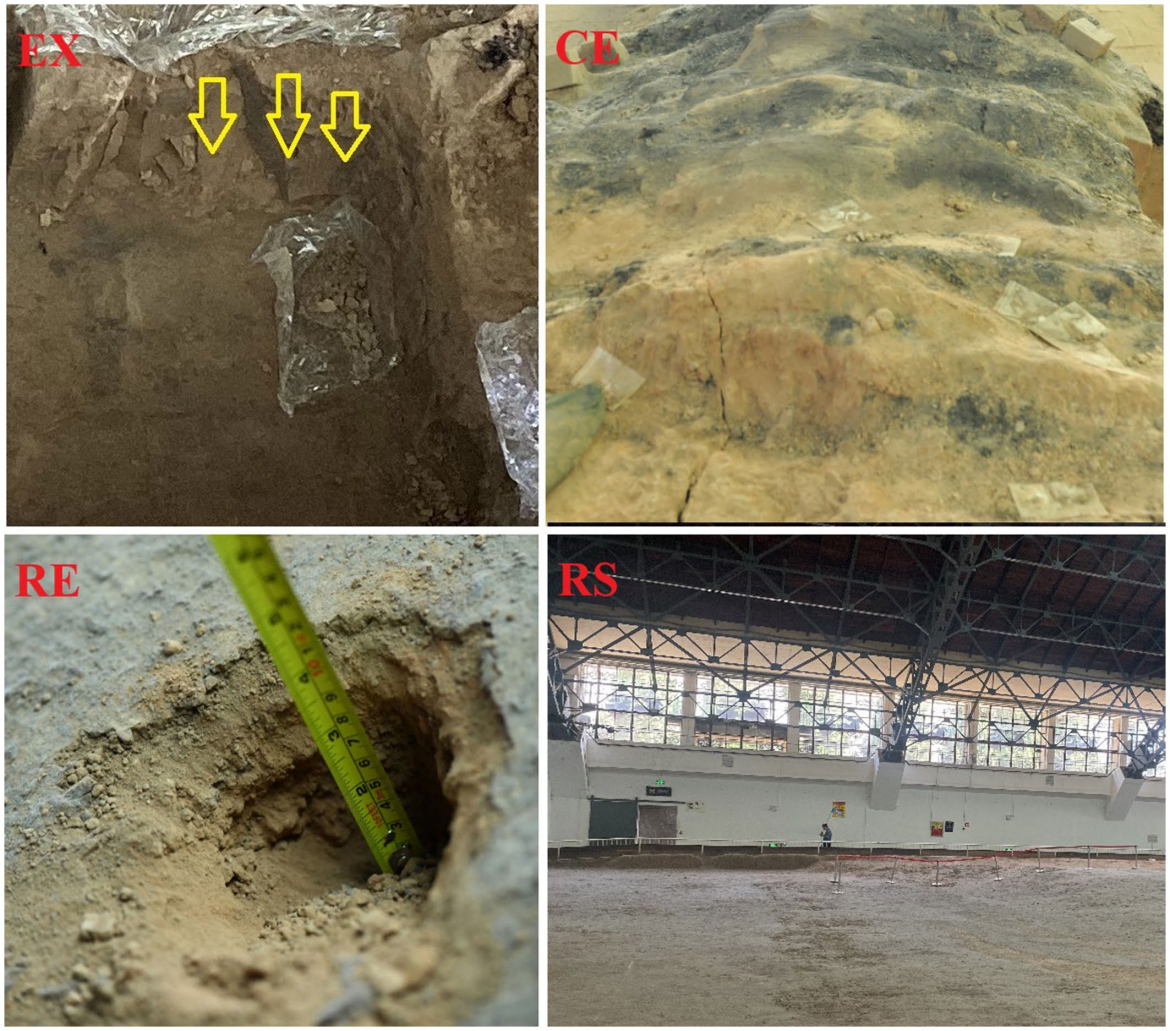
Environmental images of soil sampling locations. Excavation Area (EX), Canopy Area (CE), Restoration Area (RE), Restoration Area Surface (RS).

In July 2022, we investigated the soil at the Terra Cotta Warriors archaeological site. We divided the exhibition hall of Pit 1 of the Terra Cotta Warriors into four areas: excavation, canopy, the surface under the restoration, and the surface of the restoration. The locations are shown in Figure 1. We found a large amount of flocculent material in the restoration area, consistent with previous research results. Soil samples will be collected to analyze soil physical and chemical properties and bioindicators. A metagenomic library was established, and the species and functional diversity of each sample were analyzed. At the same time, a correlation analysis was conducted between the composition of microbial communities and environmental factors. The main culturable fungi obtained from the two samples were isolated, purified, and identified. A volatile gas fungistasis experiment was conducted to test whether allicin could inhibit the growth of these fungi. Our research provides basic data for the long-term protection of the exhibition hall of Pit 1 of the Terra Cotta Warriors in the Emperor Qinshihuang’s Mausoleum Site Museum and puts forward protection suggestions for microbial diseases. This provides relevant data on the impact of microorganisms on earthen site protection and the planning of earthen site protection strategies.

## 2 Materials and methods

### 2.1 Sample collection

Pit No. 1 at the Terracotta Warriors Museum is divided into four areas: excavation area (EX), canopy area (CE), restoration area (RE), and restoration area surface (RS). The excavation area (EX) is the pit in which the Terracotta Warriors archaeological excavations are carried out, in which the soil may come into direct contact with the Terracotta Warriors and other artifacts. The rammed earth walls in the canopy area (CE) effectively supported the huge roof of the trellis, ensuring that it would not collapse, and played an important role in the protection of the Terracotta Warriors. Scaffolding wood filled with green paste mud, with moisture, anticorrosive effect. The restoration area (RE) and the surface of the restoration area (RS) are the areas where the terracotta warriors were restored and exhibited next to the terracotta pits. The samples of RE were collected from the soil about 10 cm below the top soil layer (RS). Gray flocs were found in all four areas, and many flocs accumulated on the restoration area surface. Three parallel samples were collected from each area using sterile spoons and placed in sealed bags. Soil samples were collected from each area at specific locations, times, and temperatures, as listed in Table 1. Some were transported to the laboratory using ice bags, whereas others were transported to the laboratory using dry ice. Those transported in ice bags were stored in a 4°C refrigerator, while those transported with dry ice were stored in a − 80°C refrigerator.

**Table 1:** Environmental information of soil sample collection locations.

Sampling location	EX	CE	RE	RS
Location description	T24, G10 (West Section)	T24, Q9 (West Section)	Open space on the northwest side of the restoration area	Open space on the northwest side of the restoration area
Time	10:30	10:00	9:30	8:40
Real-time temperature	23.9°C	30.9°C	28.2°C	28.2°C
Real-time relative humidity	79.8%	56.6%	21.02%	21.02%

### 2.2 Isolation and purification of cultivable fungi(microbial isolation, cultivation, and identification)

Twelve soil samples were diluted 103 times, and the diluted soil suspension was separately applied to the PDA (Potato dextrose agar medium) medium, with three replicates for each medium of each sample. After 3 days of culture at 28°C, a single fungal colony was selected, inoculated in the new PDA medium, and cultured at 28°C for 3 days again until pure strains were obtained for subsequent experiments. All pure microorganisms were stored at −80°C.

### 2.3 Metagenomics data assembly and analyses

Total microbial genomic DNA was extracted using the DNeasy PowerSoil Kit (QIAGEN, Inc., Netherlands) following the manufacturer’s instructions. The quantity and quality of extracted DNA were measured using a NanoDrop ND-1000 spectrophotometer (Thermo Fisher Scientific, Waltham, MA, United States) and agarose gel electrophoresis, respectively. The extracted microbial DNA was processed to construct metagenome shotgun sequencing libraries with insert sizes of 400 bp using the Illumina TruSeq Nano DNA LT Library Preparation Kit. Each library was sequenced using the Illumina HiSeq X-ten platform (Illumina, United States) using the PE150 strategy at Personal Biotechnology Co., Ltd. (Shanghai, China) (Huang et al., 2021).

The raw sequencing reads were processed to obtain quality-filtered reads for further analysis. Sequencing adapters were removed from sequencing reads using Cutadapt (v1. 2. 1). Low-quality reads were trimmed using a sliding-window algorithm. Sequencing reads were aligned to the host genome using Burrow-Wheeler Aligner (BWA) to remove host contamination. Once quality-filtered reads were obtained, they were assembled *de novo* to construct a metagenome for each sample using the Iterative De Bruijn graph assembler for sequencing data with highly uneven depth. All coding regions (CDS) of the metagenomic scaffolds longer than 300 bp were predicted using MetaGeneMark. The CDS sequences of all samples were clustered using Cluster Database at High Identity with Tolerance (CD-HIT) with 90% protein sequence identity to obtain a non-redundant gene catalog. The gene abundance in each sample was estimated using soap coverage based on the number of aligned reads. The lowest common ancestor taxonomy of the nonredundant genes was obtained by aligning them against the NCBI NT database using BLASTN (e-value <0. 001). Similarly, functional profiles of non-redundant genes were obtained by annotation against the KEGG, EggNOG, and CAZy databases using the DIAMOND alignment algorithm at Shanghai Personal Biotechnology Co., Ltd. (Shanghai, China) (Huang et al., 2021).

### 2.4 Analysis of species diversity and functional diversity of microorganisms in soils of different regions

Analysis was performed using GenesCloud, a free online platform for data analysis.^1^ QIIME was used to determine the composition and abundance distribution of each sample at each classification level (domain, phylum, class, order, family, genus, and species), integrate the species annotation information of contig sequences with the abundance table in each sample, and obtain the species abundance table of each sample at each classification level. Based on the sequencing results, the composition of the microbial communities in the soil samples was analyzed at the genus level to conclude.

The common and unique functional groups of each sample were analyzed. Based on the composition spectrum of the underlying functional units annotated in each functional database for each sample, the R software was used to calculate and visually present the number of common and unique functional class groups for each sample using Venn diagrams. Principal coordinate analysis (PCoA) was used to analyze the functional beta diversity of each sample. This was done to obtain the spatial distribution characteristics of the sample functional composition and quantify the differences between the samples.

### 2.5 Analysis of the physical and chemical properties of the environmental factor and association analysis

Twelve soil samples of approximately 100 g each were sent to the Institute of Applied Ecology, Chinese Academy of Sciences, for chemical, physical, and bioindicator determination.

The soil texture and structure, water content, trace element content, heavy metal content, organic carbon content, microbial carbon content, phosphorus-related hydrolase, and carbon-related hydrolase were mainly measured as environmental factors for the correlation analysis. Soil pH (water) was determined with a glass electrode (pH 700 Bench Meter, Eutech Instruments) at a ratio of 1:2. 5. Soil total carbon (C), nitrogen (N), and sulfur (S) were measured using an automatic element analyzer (Analyzer vario MICRO cube, Elementar, Germany). The trace metals were extracted using 0. 1 M BaCl2 and determined using Inductively Coupled Plasma Optical Emission Spectrometer (ICP-OES). Microbial biomass carbon(MBC) was obtained using the chloroform fumigation direct extraction method. α-glucosidase activity (α) and β-glucosidase activity (β) were obtained by the p-nitrophenol method. Cellobiohydrolase activity (CBH), xylosidase activity, phosphomonoesterase I (ALP)and II (ACP) were determinedis obtained by the p-nitrophenylphosphate method, and phosphodiesterase (PDEs) by the bisnitrophenylphosphate method.

An environmental factor association analysis heat map was plotted using heat map tools on the GenesCloud platform.^2^ The tool was developed using the pheatmap package (V1. 0. 8), which was slightly modified to improve the layout style. The data were normalized by z-scores. The package uses popular clustering distances and methods implemented in the dist and hclust functions in R. Based on the species annotations of each sample in the database at the genus level and the obtained soil physicochemical property analysis results, the correlation between each sample species and the environment was visually presented using a heatmap.

### 2.6 Antimicrobial experiment of volatile allicin gas

The antimicrobial effect of allicin volatile gas was determined using a dichotomy plate with culturable fungi that were isolated and purified as the experimental object (Huang et al., 2019). A PDA medium was poured on one side of a split plate. After medium solidification, a fungal coating with a diameter of approximately 8 mm was applied to the culture medium. The air concentration of allicin was 625 μL/L when 50 μL allicin was added to the other side; the volume of the 90 mm culture dish was about 80 mL. An equal amount of sterile distilled water was used as a negative control to observe the inhibitory effect.

### 2.7 Bacterial growth culture and heavy metal ion stress growth culture

During the excavation of the Terracotta Warriors, many copper weapons were unearthed, so there are high levels of copper in the soil, especially in the excavated areas(the average value is 48.760 mg/kg). Macrogenomic sequencing analysis showed that *Cupriavidus* were found in the excavation area where a large number of copper artifacts had been buried. Therefore, we carried out further analyses of the *Cupriavidus alkaliphilus*.

For the particular bacterium *C. alkaliphilus*, which was isolated and obtained from the excavation area and account for 0. 15% of species diversity, even in the excavation areas the percentage reaches 6. 75%. We performed the growth curve of this strain. One ring of activated *C. alkaliphilus* plaque was inoculated into the 50 mL LB liquid medium, incubated at 37°C, 180 rpm for 16 h, and reserved for use. The sterilized LB liquid medium was divided into 18 100 mL sterilized triangular bottles, each filled with 20 mL of liquid, sealed with air-permeable sealing film, and labeled and numbered; one bottle was labeled as the control and stored at 4°C, and the other 17 bottles were labeled as 0, 2, 4, 6, 8, 10, 12, 14, 16, 18, 20, 22, 24, 26, 28, 30, and 32 h. Each bottle was inoculated with the above-mentioned bacterial seed solution at an inoculum of 10% (V/V), and shake cultivation was performed for 16 h at 37°C and 180 rpm and the corresponding bottles were taken out according to the labels at the corresponding time and then preserved in the refrigerator at 4°C for the measurement of the OD_600_ nm values by photoelectrophoresis turbidimetry. Three parallel groups were established at each time point. The measurements were performed until a plateau was reached.

The LB liquid medium was prepared by adding CuSO_4_-5H_2_O so that the Cu^2+^ concentration gradient was 5, 7. 5, 10, 20, 25, and 30 mg/L (in terms of pure Cu^2+^), and 17 triangular flasks of each concentration were labeled as 0, 2, 4, 6, 8, 10, 12, 14, 16, 18, 20, 22, 24, 26, 28, 30, and 32 h. In addition, the LB liquid medium without any heavy metal ions was used as the control, and the pre-cultured bacterial solution was inoculated into the above medium at an inoculum of 10% (V/V) and placed on a shaker (180 rpm) at a constant temperature (37°C) for the shake flask culture. The corresponding flasks were taken out at the corresponding times according to their labels and stored in the refrigerator at 4°C. Three parallel groups were established at each time point. Measurements were taken until a plateau was reached.

### 2.8 Measuring bacterial growth by the photo turbidimetric method and plotting growth curves and growth histograms

After sampling the bacterial growth culture solution, the wavelength of the UV–visible spectrophotometer was adjusted to 600 nm. The OD_600nm_ value of the bacterial solution of *C. alkaliphilus* at different incubation times was determined, and the average value of each sample was taken thrice. The range of the spectrophotometer was 0. 0000–3. 0000; if the mass concentration of the bacterial suspension was too large, it was diluted at appropriate times, and the incubation time, dilution times, and OD_600nm_ value were recorded. The incubation time of *C. alkaliphilus* served as the horizontal coordinate, the average OD_600nm_ value of the measured bacterial solution was used as the vertical coordinate, and the growth curve of *C. alkaliphilus* was drawn. Using the Cu^2+^ concentration as the horizontal coordinate and the OD_600nm_ value as the vertical coordinate, the histogram of the maximum amount of growth of Cu^2+^-*C. alkaliphilus* was plotted.

## 3 Results

### 3.1 Cultivable fungi isolation, cultivation, and identification

**Figure 2:**
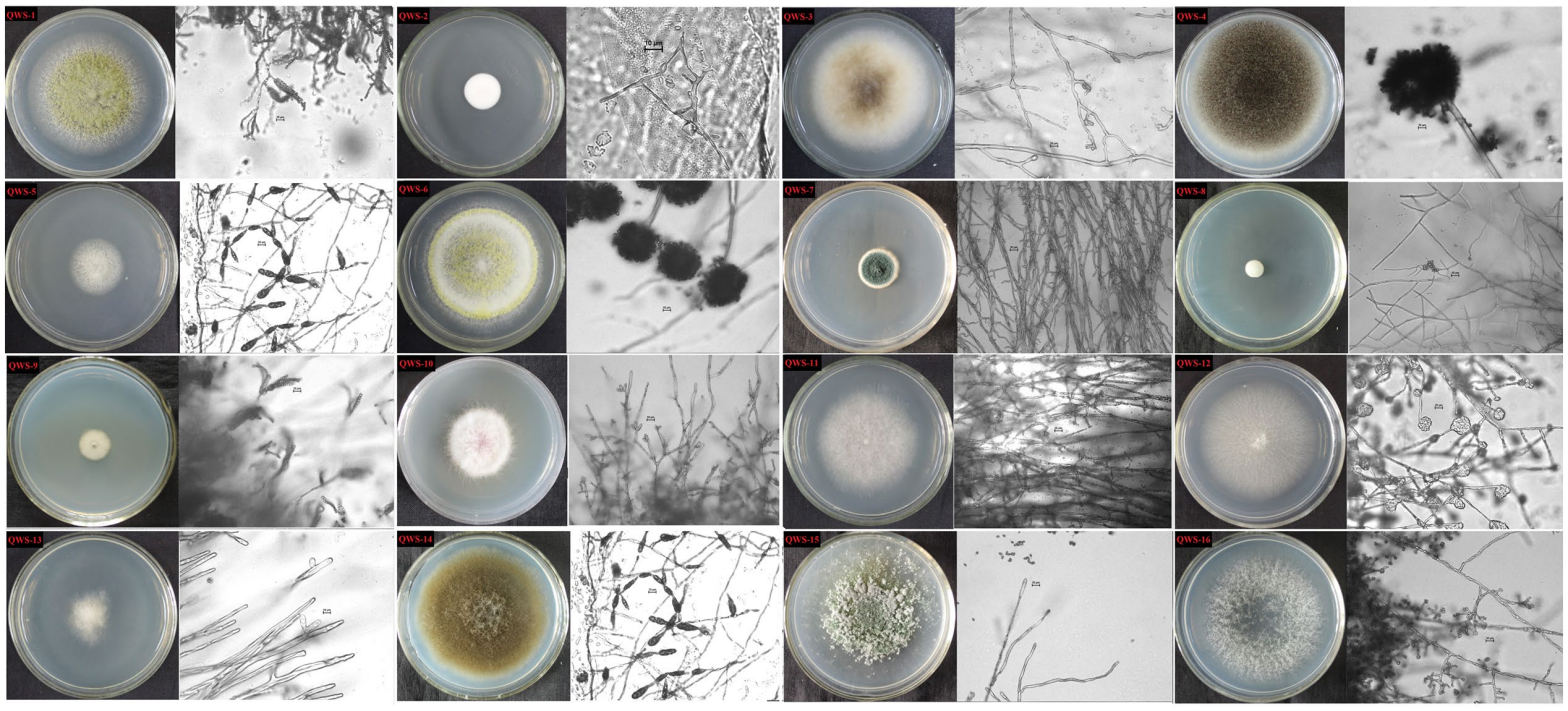
Plate photos and micrographs of isolated and purified fungi (Inner diameter of the medium is 5cm, The scale in the micrograph is 10μm).

In July 2022, we collected three soil samples from the excavation, canopy, restoration area, and restoration area surface in the exhibition hall of the Terra Cotta Warriors Burial Pit No. 1 of the Emperor Qinshihuang’s Mausoleum Site Museum. A total of 12 samples were collected. Following separation and purification, 28 cultivable fungi were identified and classified. Of these, 16 were identified in previous experiments (Qiang et al., 2021) and may be the main pathogenic fungi, as shown in Table 2. The plate photographs and photomicrographs of the fungi isolated from the soil are presented in Figure 2.

**Table 2:** Identification results of cultivable fungi isolated and purified from soil samples.

QWS-1	*Penicillium chrysogenum*	100.00%	MK841451.1
QWS-2	*Simplicillium aogashimaense*	100.00%	MT860448.1
QWS-3	*Penicillium corylophilum*	99.89%	MH876147.1
QWS-4	*Aspergillus niger*	99.82%	KT192262.1
QWS-5	*Aspergillus versicolor*	99.80%	KP940477.1
QWS-6	*Aspergillus flavus*	99.42%	MT509808.1
QWS-7	*Aspergillus sydowii*	99.69%	KJ524908.1
QWS-8	*Aspergillus puulaauensis*	99.68%	AP024448.1
QWS-9	*Alternaria alternata*	99.69%	KT207688.1
QWS-10	*Fusarium proliferatum*	98.27%	MG274295.1
QWS-11	*Fusarium fujikuroi*	99.90%	CP023090.1
QWS-12	*Fusarium solani*	99.77%	MT579869.1
QWS-13	*Fusarium incarnatum*	100.00%	MT560229.1
QWS-14	*Talaromyces funiculosus*	99.77%	MH876669.1
QWS-15	*Trichoderma longibrachiatum*	100.00%	MT520608.1
QWS-16	*Trichoderma harzianum*	99.27%	MN611195.1

### 3.2 Fungal community analysis

**Figure 3:**
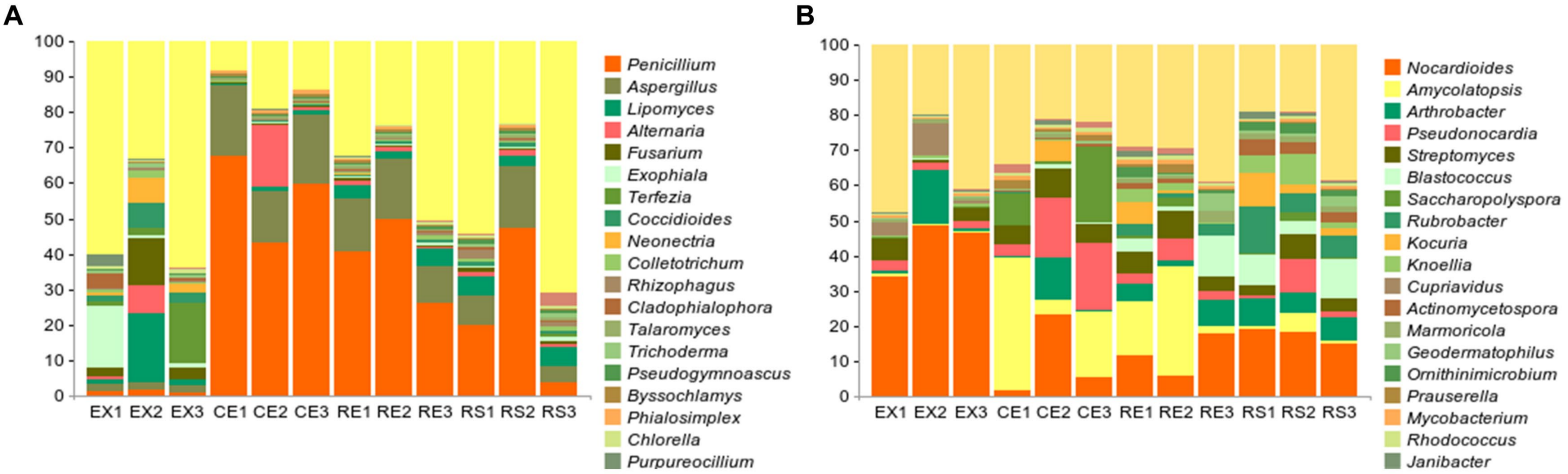
Microbial diversity results of soil samples (**A** represents fungal genus-level diversity results, **B** represents bacterial genus-level diversity results).

The microbial diversity results obtained from the above-mentioned 12 samples were analyzed. The results are shown in Figure 3, where they rank among the top 20 in terms of genus-level abundance. *Penicillium*, *Aspergillus*, *Lipomyces*, *Alternaria*, and *Fusarium* accounted for the largest proportion. Among the 20 genera present in the largest proportions, *Penicillium*, *Aspergillus*, *Alternaria*, *Fusarium*, *Talaromyces*, and *Trichoderma* were obtained in isolation and purified. The analysis of the microbial community composition in the 12 soil samples showed that the top ten microorganisms in abundance were all bacteria, as shown in the Figure 3. Among these, *Nocardioides*, *Amycolatopsis*, *Arthrobacter*, *Pseudonocardia*, and *Streptomyces* accounted for the largest proportion. The abundance of fungi and bacteria in the soil suggests the potential harm to soil sites and even to the Qin Terracotta Warriors.

**Figure 4:**
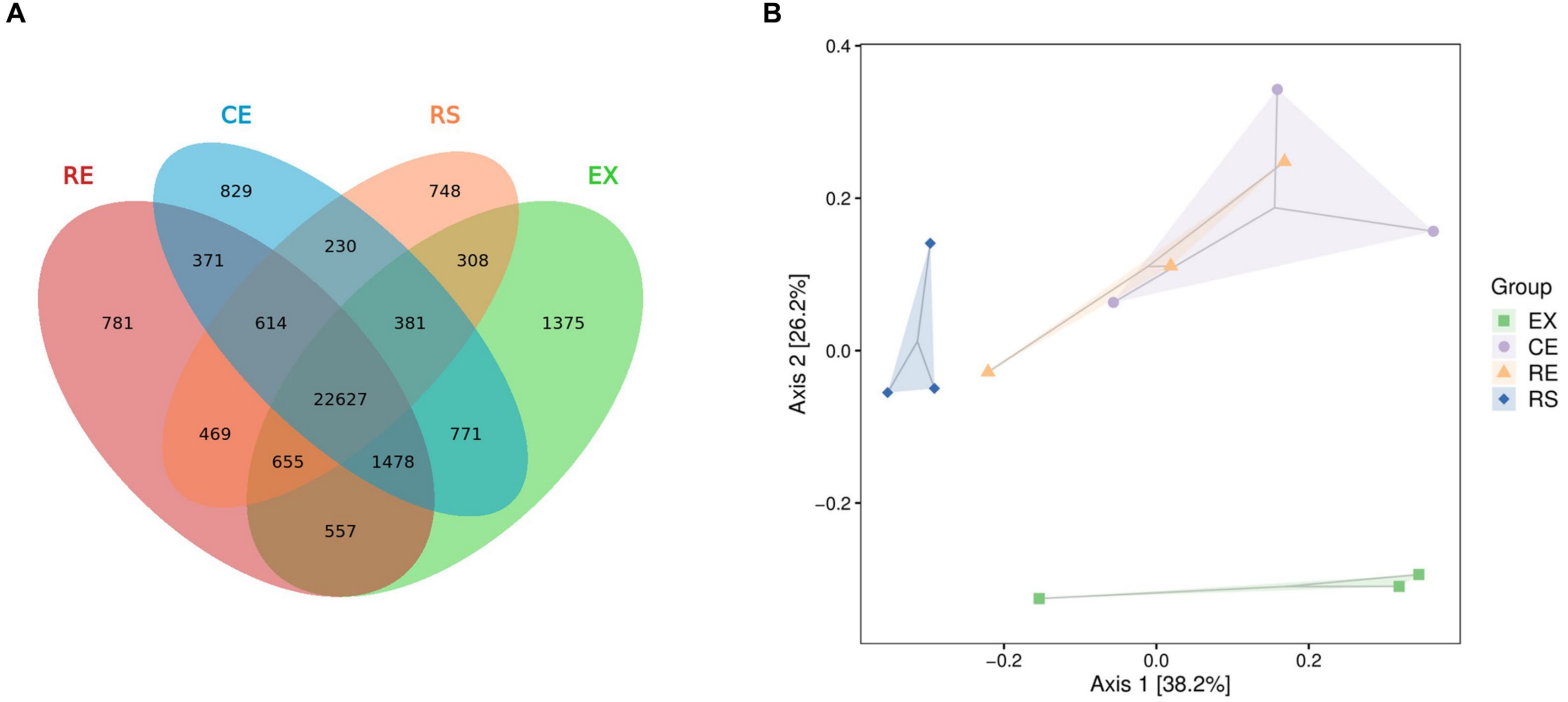
Analysis of the number and beta diversity of common and unique species groups in soil samples from four regions. (**A** is the Venn diagram, **B** is the PCoA diagram).

Venn plots of the number of common and unique species groups in the collected samples from the four regions were drawn, and PCoA analysis on their beta diversity was performed to quantify the differences between the samples. From Figure 4 shows that the excavation area contains more unique species, whereas the excavation area (EX) and canopy area (CE) contain more of the same species. There were significant differences in species between the excavation area (EX) and surface area (RS) of the restoration area and other groups. The species group differences between the two samples in the canopy area (CE) and restoration area (RE) were relatively small, and the similarity was relatively high.

### 3.3 Analysis of the functional diversity of microorganisms

**Figure 5:**
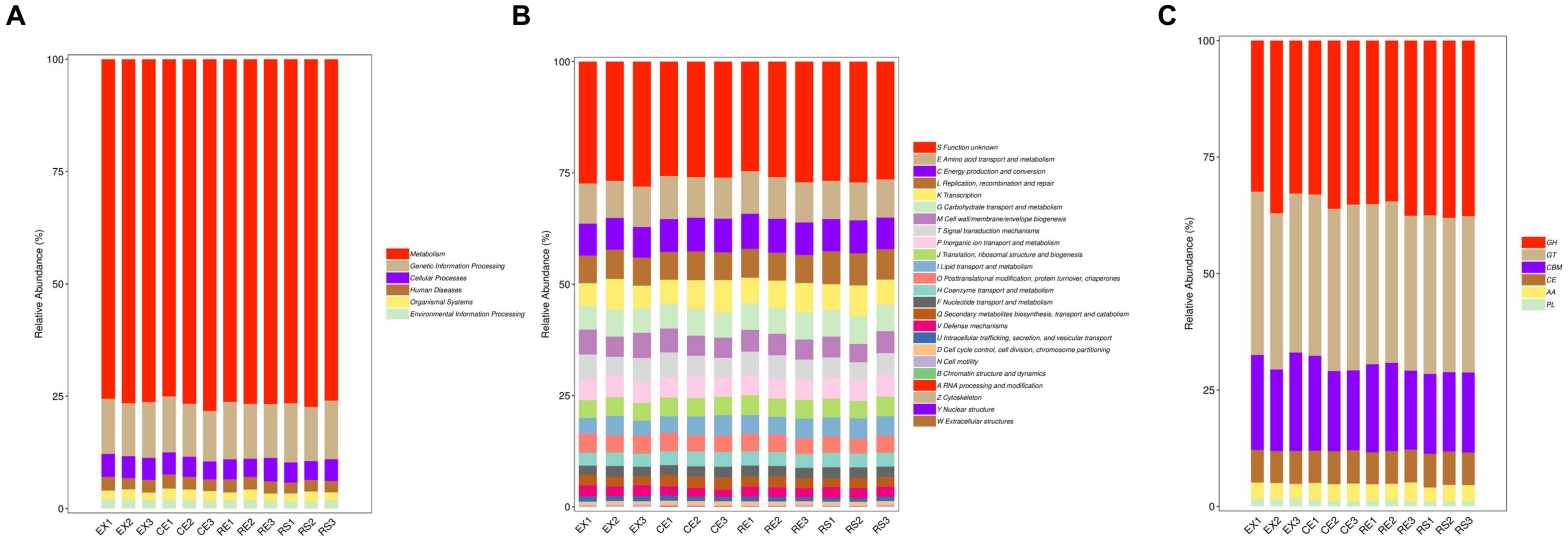
Bar chart drawn based on the composition spectrum of functional units annotated in the KEGG, EggNOG, and CAZy functional databases for each sample (**A** is the KEGG database result, **B** is the EggNOG database result, and **C** is the CAZy database result).

The KEGG Metabolic Pathway Database is the core database of KEGG. This database systematically categorizes metabolic pathways into seven major categories: metabolism, genetic information processing, environmental information processing, cellular processes, organic systems, human diseases, and drug development. By integrating the annotated the KO database of soil microorganisms with the gene abundance matri, the top three pathways ranked in abundance were found to be related to metabolism, genetic information processing, and cellular processes. The corresponding KO abundance of each protein was obtained, as shown in Figure 5A.

The EggNOG database is managed by the European Molecular Biology Laboratory, which uses the Smith-Waterman comparison algorithm to annotate the functions of orthologous groups of genes. A relative abundance diagram is shown in Figure 5B. The top three are amino acid transportation and metabolism, energy production and conversion, replication, recombination, and restoration area. Using the EggNOG annotation of soil sample microorganisms, the bias and composition of microbial protein production in excavated soil can be analyzed, providing data support for various mechanisms and functions of research.

The CAZy database focuses on carbohydrate-active enzymes that degrade, modify, and generate glycosidic bonds. This is a professional database used to study related enzymes. Based on the similarity of amino acid sequences in protein domains and their corresponding functions, CAZy divides these carbohydrate-active enzymes into six major functional protein modules: glycoside hydrolases, glycosyl transferases, polysaccharide lyases, carbohydrate esterases, auxiliary oxidoreductases, and carbohydrate-binding modules. A relative abundance diagram is shown in Figure 5C. The top three modules were glycoside hydrolase, glycosyltransferase, and carbohydrate-binding modules. By annotating the CAZy database of soil microorganisms in excavation pits, the bias and composition of microbial carbohydrate enzyme systems can be fully analyzed, providing data support for the study of pathways such as microbial degradation and metabolism.

**Figure 6:**
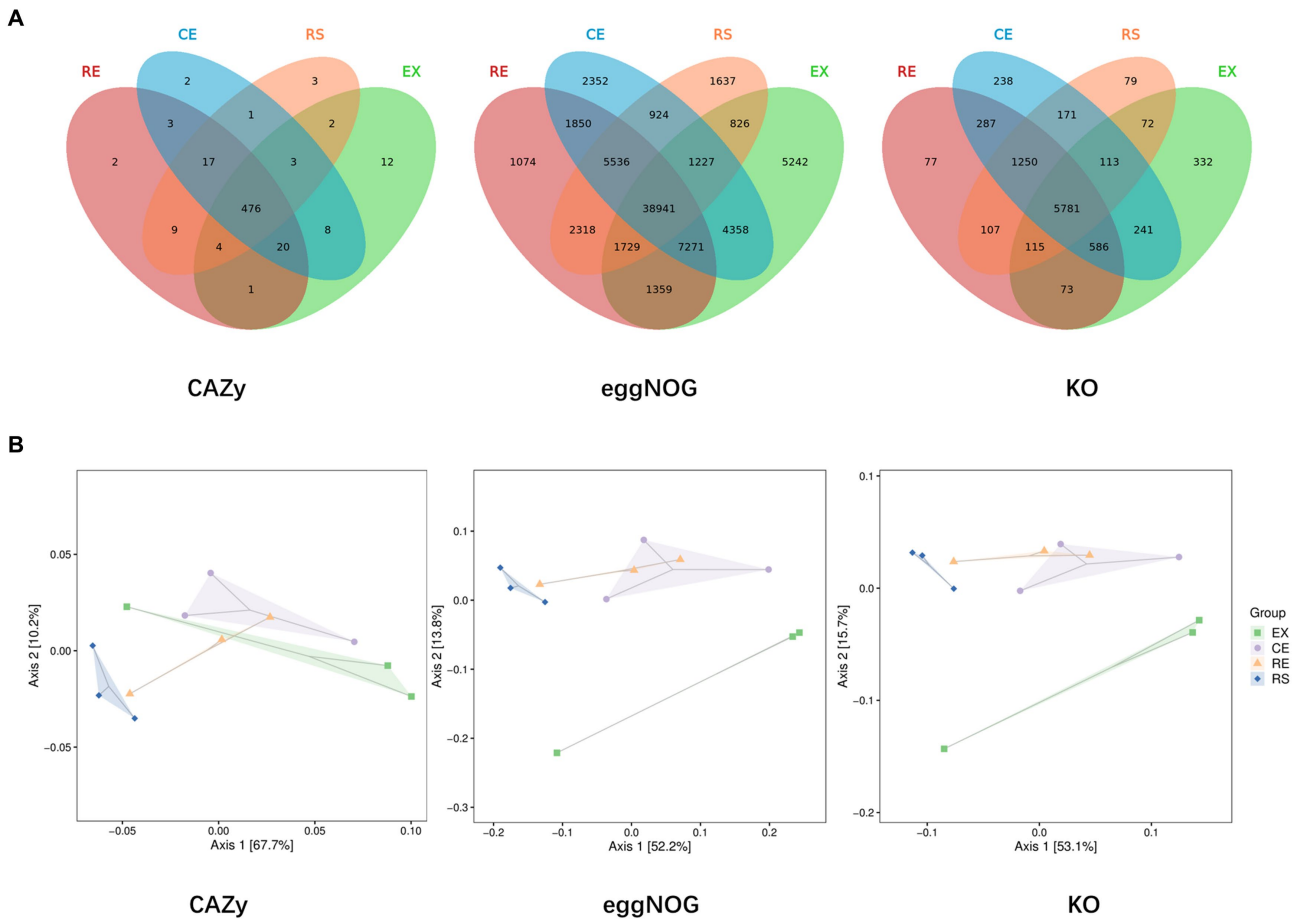
Analysis of the number and beta diversity of common and unique functional groups in soil samples from four regions. (**A** is the Venn diagram obtained from three databases, and **B** is the PCoA diagram obtained from three databases).

Venn plots of the number of common and unique functional groups in the collected samples from the four regions were drawn, and PCoA analysis on their beta diversity was performed to quantify the differences between the samples, as shown in Figure 6. The excavation area contains a relatively large number of unique species and functional components. The species group differences between the two samples in CE and RE were relatively small, and the similarity was relatively high.

### 3.4 Correlation analysis between microorganisms and soil physical and chemical properties

**Figure 7:**
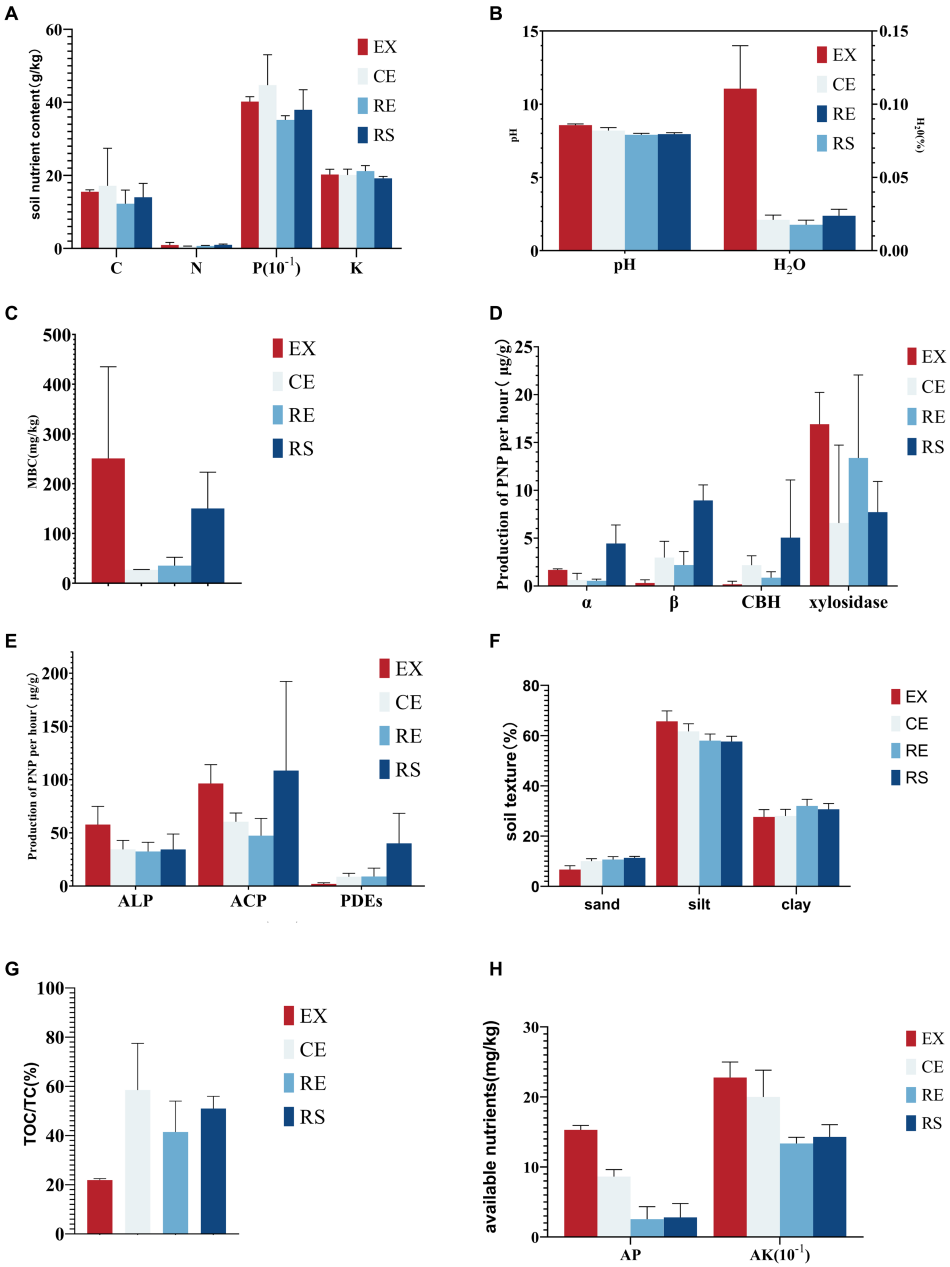
Results of the chemical, physical, and bioindicator determination of soil samples (**A** is the content of soil macroelements, **B** is the soil water content and pH, **C** is the microbial biomass, **D** is the activity of soil carbon-related hydrolase, **E** is the activity of soil phosphorus-related hydrolase, **F** is the soil texture, **G** is the proportion of soil organic carbon to the total carbon content, and **H** is the content of soil available nutrients).

The soil macroelement content was the lowest in the remediation area, but no significant differences among the sampling points were found, as shown in Figure 7A. The soil moisture content, pH, and microbial biomass carbon content in the excavation area were significantly higher than those at the other sampling points, indicating that soil conditions, such as humidity and pH, are more conducive to microbial growth in the area, as shown in Figures 7B,C. The activity of soil carbon-related hydrolase is the highest in the surface sampling area. In particular, α- and β-glucosidases indicate that the hydrolysis of soil organic matter in this area increases, which is not conducive to the stability of soil mass, as shown in Figure 7D. This result was consistent with the metagenomic sequencing results from the CAZy database. Similar to carbon-related hydrolase, the activity of soil phosphorus-related hydrolase was the highest at the surface sampling point, followed by the excavation area, which proves that the hydrolysis of soil organic matter in this area increased, which is not conducive to land stability, as shown in Figure 7E. The proportion of silt particles in the soil at each sampling point was highest, followed by those of sand and clay particles. The proportion of silt particles in the soil of the excavation area was the highest, whereas that of clay particles decreased. The risk of soil erosion in this area was high, as shown in Figure 7F. Available nutrients in the soil are crucial for the growth of soil microorganisms. There was no significant difference in the alkali-hydrolyzed nitrogen content of the soil at each sampling point. The soil of the excavation and canopy area had the highest contents of available phosphorus and potassium, which could provide effective nutrients for microbial activities, as shown in Figure 7G. Due to the alkalinity of the soil, there is a carbonation reaction when adding acid, which increases the determination of soil organic carbon content. The results showed that the total carbon content of the soil was 11. 88–15. 27%, whereas the organic carbon content was only 3. 39–11. 32%. In particular, in the excavation area, the organic carbon content was only 21. 9% of the total carbon content, as shown in Figure 7H.

**Figure 8:**
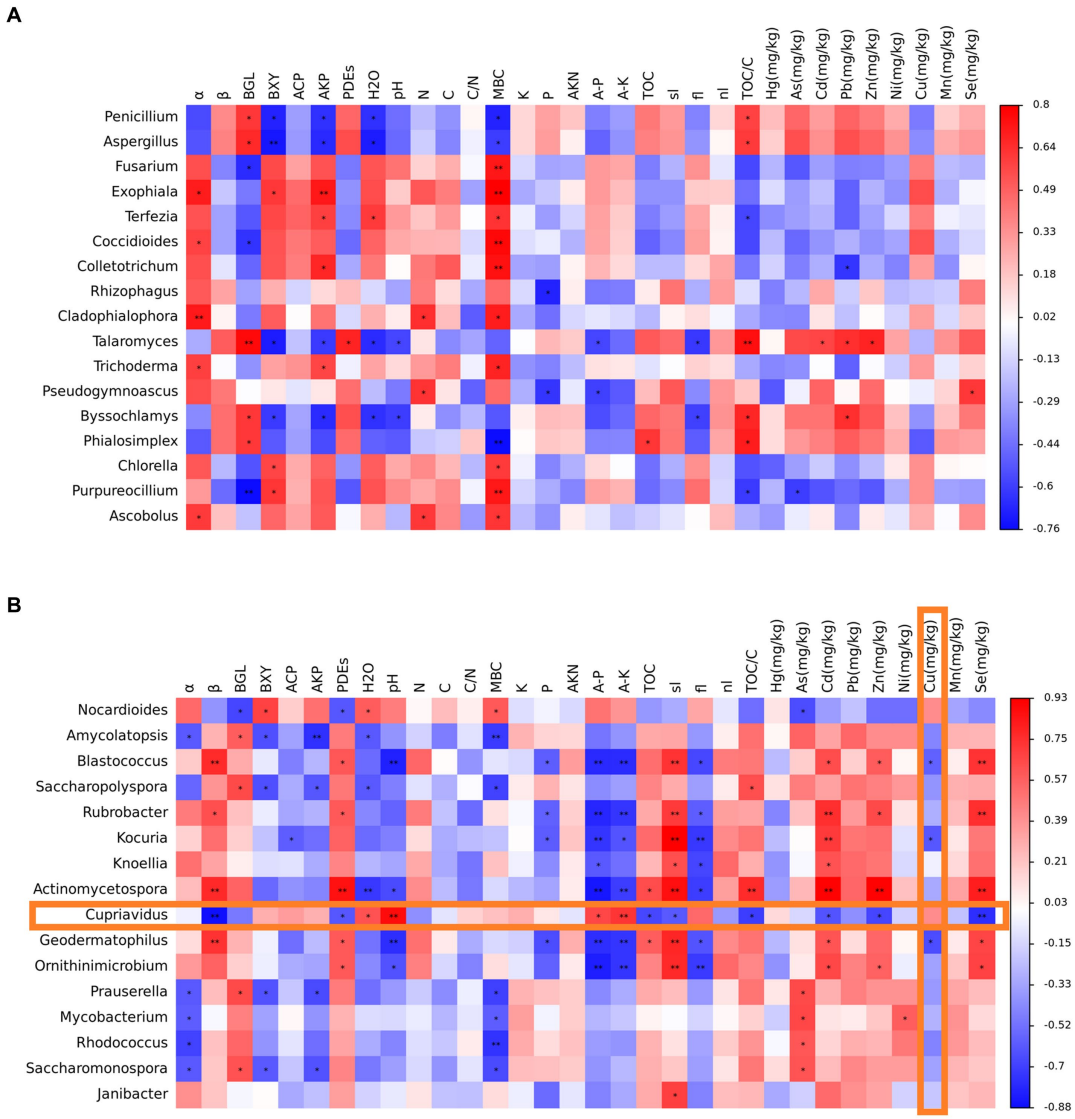
Analysis results of the correlation between dominant fungi and dominant bacteria with relevant environmental factors (**A** represents fungal results, **B** represents bacterial results, *: *p* < 0.05, **: *p* < 0.01).

The following environmental factors most closely related to microbial growth activities were screened: α- glucosidase activity (α) and β-glucosidase activity (β). The correlation between water content (H_2_O), soil pH (pH), microbial biomass carbon (MBC), available phosphorus (P), and available potassium (K) was analyzed with the genera of dominant fungi and bacteria, and a heat map was drawn (Figure 8). Soil α-glucosidase activity, cellobiose hydrolase, alkaline phosphomonoesterase, microbial biomass carbon, and organic carbon in the total carbon ratio significantly correlated with the content of dominant fungi and were mostly positively correlated. There was a significant and mostly negative correlation between the soil moisture content and dominant fungal content. *Penicillium*, *Aspergillus*, *Talaromyces*, *Filamentomyces*, and *Phialosimplex* differed from the other genera. Soil α-glucosidase activity, available phosphorus, available potassium, water content, microbial biomass carbon, and powder and copper content significantly correlated with the content of dominant bacteria, with most of them being negatively correlated. Soil β-glucosidase activity, phosphodiesterase, sand content, cadmium ion concentration, and selenium ion concentration significantly correlated with the content of dominant bacteria, with most of them being positively correlated. The relationship between *Nocardioides* and *Cupriavidus* and soil properties was more specific. The correlation analysis of soil property factors with dominant fungi and bacteria provided data support for analyzing the function of microorganisms in the soil of the Emperor Qinshihuang’s Mausoleum Site Museum and their impact on soil sites.

### 3.5 Antimicrobial experiment of allicin volatile gas

**Figure 9:**
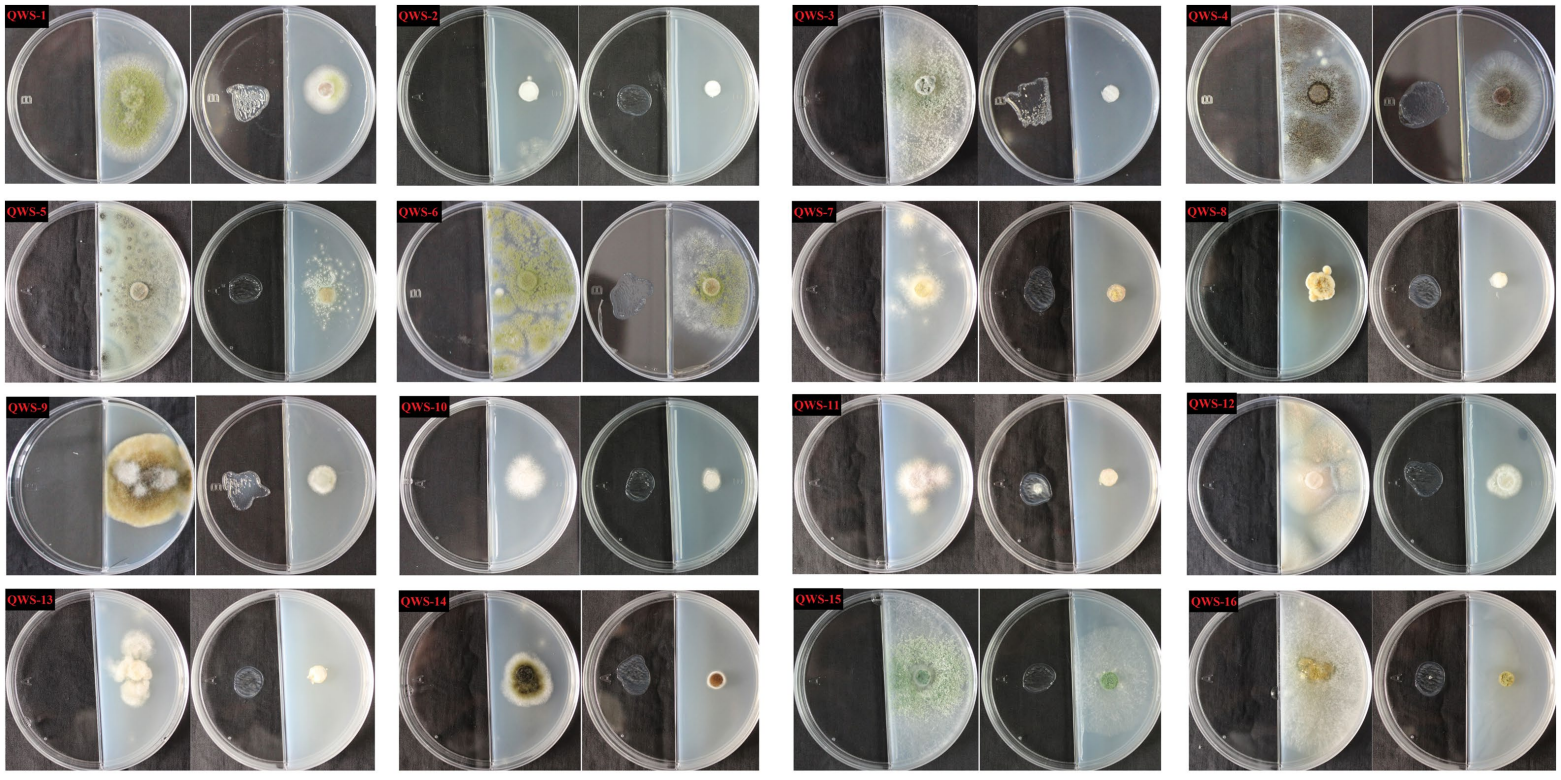
Antifungal effect of volatile allicin gas determined by dichotomy (Inner diameter of the medium is 9 cm) Fungal moss is 8mm in diameter).

Dichotomy was used to determine the antifungal effect of allicin volatile gas. Sterile distilled water was used as the negative control. The inhibitory effect was observed when the air concentration of allicin was 625 μL/L. As shown in Figure 9, under normal growth conditions of fungal hyphae in the negative control group, allicin gas had a potent inhibitory effect on the growth of the most culturable fungal hyphae. Allicin gas had a potent inhibitory effect on 12 types of fungi.

### 3.6 Growth curve and growth volume histogram of *Cupriavidus alkaliphilus*

**Figure 10:**
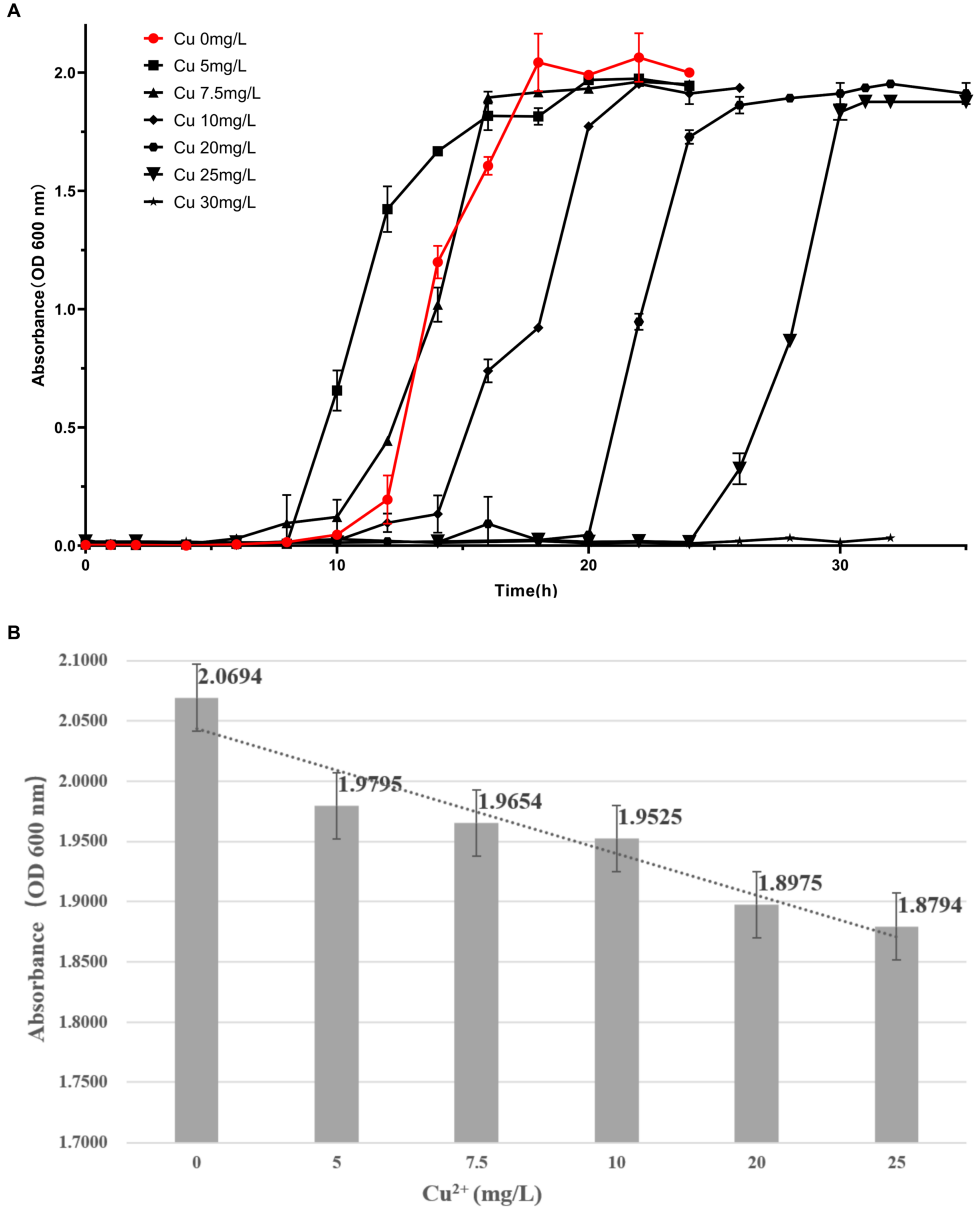
Growth curves and histograms of copper ion stress in the growth of Cupriavidus alkaliphilus.

The culture of *C. alkaliphilus* was carried out by traditional shaking cultivation, and its growth was measured by the phototurbidimetric method at OD_600_ nm using an uninoculated pure medium as a negative control. The growth curves and amounts of *C. alkaliphilus* at different Cu^2+^ concentrations are shown in Figure 10. With an increase in Cu^2+^ concentrations, the acclimatization period of *C. alkaliphilus* was significantly prolonged, and its growth in the culture containing Cu^2+^ was lower than that in the culture without copper ions.

## 4 Discussion

We analyzed the physical and chemical properties and bioindicators of soil samples from earthen sites in the exhibition hall of the Terra Cotta Warriors burial pit No. 1 of the Emperor Qinshihuang’s Mausoleum Site Museum. A metagenomic library was established, the species diversity of the microbial community was analyzed, and functional diversity was predicted. Simultaneously, we analyzed the correlation between the microbial community composition and environmental factors. This study provides basic data for the prevention and treatment of microbial diseases in soil sites in the exhibition hall of the Terra Cotta Warriors burial pit No. 1 of the Emperor Qinshihuang’s Mausoleum Site Museum and is of great significance for protecting the soil site museum.

### 4.1 Analysis of microbial diseases in earth site of Terra Cotta Warriors Pit No.1

The mausoleum of Emperor Qin Shi Huang was built more than 2, 000 years ago. The Terra Cotta Warriors were discovered in March 1974, and excavations began in July. The No. 1 Pit has been excavated three times on large scales (Ma et al., 2016). The exhibition hall of the no. 1 Pit is the earliest semi-open exhibition hall built and opened to the public at the Emperor Qinshihuang’s Mausoleum Site Museum. The exhibition hall is a steel-frame building. Apart from the necessary measures to avoid sunlight and rain, there are no other environmental protection measures. Sunlight can directly illuminate the site hall through glass windows, causing significant changes in temperature and humidity in areas exposed to sunlight, may also cause the growth of some photosynthetic microorganisms (Dexing, 2013; Binyaseen, 2022). Owing to the large space in the exhibition hall, effective temperature and humidity controls cannot be implemented. With an increasing number of tourists and the entry of archaeological excavation personnel, many microorganisms and various coarse and fine particles have gathered in the exhibition hall of Pit 1. The aggregation of flocs further accumulates dark sediments on the surface of the earthen site, leading to further microbial contamination. Early scholars conducted targeted research. Zhang et al. speculated that the surface sediment of the site and cultural relics came from clothing fibers worn by many tourists. Li et al. suggested that dust discharge from nearby industries, power plants, and civilian furnaces also adds to the dust from Pit 1. Furthermore, several poplar and willow trees are planted around the South Ring Road of Pit 1 (Li Hua and Du Weisha, 2019). In the canopy and restoration areas, especially on the surface of the restoration area, these flocs can be observed to gather in large quantities, whereas less flocs accumulates in the excavation area, because of its low elevation and state of excavation. Therefore, PCoA revealed that the species composition of the excavation area differed significantly from that of the other three areas.

### 4.2 Correlation between dominant fungi and bacteria in Terra Cotta Warriors Pit No. 1 and environmental factors

Using heat maps, the GenesCloud tool was used to visualize the correlation between dominant species and environmental factors in each sample. Because all four areas are located in the same exhibition hall, some of the physical properties of the soil are relatively similar. In our study, α-glucosidase activity (α), β-glucosidase activity (β), the water content, soil pH, microbial biomass carbon, available phosphorus, and available potassium are closely related to microbial growth activities. The relationship between *Cupriavidus* and soil properties was more specific. Many copper weapons were unearthed during the excavation of the Terra Cotta Warriors; therefore, there was high copper content in the soil, especially in the excavation area. *Cupriavidus* sp. was identified as a *Cupriavidus alkaliphilus* in later identification experiments, and it has genes for resistance to nickel, zinc, arsenate, chromate, cobalt, cadmium, and copper, as well as genes for the production of secondary metabolites, such as bacteriocins, non-ribosomal synthesizing peptides, iron carriers, and polyketides (Rojas et al., 2016). A large group of genes essential for resisting and detoxifying several heavy metals has also been identified (Kutralam-Muniasamy and Pérez-Guevara, 2019). Several genes are involved in the resistance to and detoxification of heavy metals in copper-greedy bacteria. Therefore, *C. alkaliphilus* can potentially harm the metal cultural relics unearthed in the burial pits of the Terra Cotta Warriors.

### 4.3 Possible potential hazards of *Cupriavidus alkaliphilus on copper artifacts*

In our experiments, *C. alkaliphilus* was cultured using media containing different concentrations of Cu^2+^. When lower Cu^2+^ were present, *C. alkaliphilus* had a shorter logarithmic period of growth and a relatively faster growth rate. The acclimatization period of *C. alkaliphilus* appeared to be significantly prolonged with increasing concentrations of Cu^2+^. The growth level of *C. alkaliphilus* was lower in cultures containing Cu^2+^ stress compared to those without Cu^2+^ ions. The presence of *C. alkaliphilus* could potentially be harmful to metallic artifacts excavated from the burial pits of the Terracotta Warriors.

### 4.4 The application value of allicin as an antibacterial agent in the protection of cultural relics

Choosing plant-based antifungal agents from actual plants is safer and more environmentally friendly. In recent years, domestic and international research has revealed multiple known and novel structural types of active plant-derived antibacterial ingredients. It is ideal for developing plant-based antibacterial agents and has received increasing attention from scholars. Allicin (C_6_H_10_OS_2_) is an organosulfur compound with the scientific name of diallyl thiosulfinate, is the main bioactive component of garlic and exhibits broad-spectrum antibacterial and antifungal activity. In this study, we used the dichotomy method to determine the antifungal effect of volatile allicin gas, which has a potent inhibitory effect on the mycelial growth of most fungi. However, owing to the odor of allicin itself, we still need to further improve this method by reducing the concentration of allicin. However, because allicin has a potent inhibitory effect on the mycelial growth of most fungi, it can be regarded as a reference for the prevention of microbial diseases in soil sites and cultural relics.

In the different areas of the exhibition hall of Pit 1, there are terracotta warriors that are excavated, restored and conserved. Therefore, the dominant fungi found in the earthen ruins may be the cause of microbial damage to the precious cultural relics of the Terra Cotta Warriors, with potential risks. Therefore, fungi discovered at soil sites must be taken seriously by conservationists. This study is of great significance for the protection of the Terra Cotta Warriors and other precious cultural relics in the Emperor Qinshihuang’s Mausoleum Site Museum.

## 5 Conclusion

The problem of microbial diseases in the earthen sites in the exhibition hall of burial pit No. 1 of the Terra Cotta Warriors in the Emperor Qinshihuang’s Mausoleum Site Museum is cannot be ignored. The microbial diseases in different areas of the exhibition hall differ because of the different environments. The main pathogenic fungi in the earthen sites may cause microbial diseases that affect essential cultural relics such as the Terra Cotta Warriors. *Cupriavidus alkaliphilus* is abundant in the soil of the excavation areas of exhibition halls and may pose a potential hazard to excavated metallic artifacts. Allicin is expected to become an effective bacteriostatic agent to prevent cultural relics, such as the earthen sites and Terra Cotta Warriors, from being persecuted by disease microorganisms. This study provides basic data on the microbiological problems of earthen sites in the exhibition hall of the Terra Cotta Warriors Burial Pit No. 1 in the Emperor Qinshihuang’s Mausoleum Site Museum, which is of great significance for the protection of the sites as well as for the protection of the Terra Cotta Warriors and other precious cultural relics in the museum.

## Data availability statement

The raw sequence data (amplicon and metagenomic datasets) were deposited into the NCBI Sequence Read Archive (SRA) database BioProject (Accession Number: PRJNA1047174).

## Author contributions

CW: Data curation, Formal analysis, Methodology, Software, Validation, Writing – original draft, Writing – review & editing. LH: Methodology, Software, Validation, Writing – review & editing. NJ: Data curation, Software, Validation, Writing – review & editing. YuW: Conceptualization, Methodology, Investigation, Writing – original draft, Writing – review & editing. XM: Investigation, Writing – review & editing. PZ: Investigation, Writing – review & editing. YX: Investigation, Writing – review & editing. YuaW: Data curation, Writing – review & editing. CC: Methodology, Validation, Writing – review & editing. XY: Software, Validation, Writing – review & editing. QL: Project administration, Supervision, Writing – review & editing. JP: Funding acquisition, Project administration, Supervision, Writing – review & editing.

## Footnotes

^1^ https://www.genescloud.cn

^2^ https://www.Genescloud.cn
